# When to stop? Decision-making when children’s cancer treatment is no longer curative: a mixed-method systematic review

**DOI:** 10.1186/1471-2431-14-124

**Published:** 2014-05-13

**Authors:** Edith Valdez-Martinez, Jane Noyes, Miguel Bedolla

**Affiliations:** 1 Health Research Council of the Mexican Institute of Social Security, Centro Medico Nacional Siglo XXI. Av. Cuauhtemoc # 330. Col. Doctores. C.P, Mexico 06720 D,F, Mexico; 2 School of Healthcare Sciences, Bangor University, Bangor LL57 2EF, UK; 3 Policy Studies Centre of the University, San Antonio TX, USA

**Keywords:** Cancer, Palliative care, Children, Young people, Parents, Professional, End-of-life, Decision-making, Systematic review

## Abstract

**Background:**

Children with cancer, parents, and clinicians, face difficult decisions when cure is no longer possible. Little is known about decision-making processes, how agreement is reached, or perspectives of different actors. Professionals voice concerns about managing parental expectations and beliefs, which can be contrary to their own and may change over time. We conducted the first systematic review to determine what constitutes best medico-legal practice for children under 19 years as context to exploring the perspectives of actors who make judgements and decisions when cancer treatment is no longer curative.

**Methods:**

Theory-informed mixed-method thematic systematic review with theory development.

**Results:**

Eight legal/ethical guidelines and 18 studies were included. Whilst there were no unresolved dilemmas, actors had different perspectives and motives. In line with guidelines, the best interests of the individual child informed decisions, although how different actors conceptualized ‘best interests’ when treatment was no longer curative varied. Respect for autonomy was understood as following child/parent preferences, which varied from case to case. Doctors generally shared information so that parents alone could make an informed decision. When parents received reliable information, and personalized interest in their child, they were more likely to achieve shared trust and clearer transition to palliation. Although under-represented in research studies, young people’s perspectives showed some differences to those of parents and professionals. For example, young people preferred to be informed even when prognosis was poor, and they had an altruistic desire to help others by participating in research.

**Conclusion:**

There needs to be fresh impetus to more effectively and universally implement the ethics of professionalism into daily clinical practice in order to reinforce humanitarian attitudes. Ethical guidelines and regulations attempt to bring professionals together by articulating shared values. While important, ethics training must be supported by institutions/organizations to assist doctors to maintain good professional standards. Findings will hopefully stimulate further normative and descriptive lines of research in this complex under-researched field. Future research needs to be undertaken through a more deliberative cultural lens that includes children’s and multi-disciplinary team members’ perspectives to more fully characterize and understand the dynamics of the decision-making process in this specific end-of life context.

## Background

Decision-making at its best combines the highest quality research evidence on the benefits and risks of various treatments, doctor clinical expertise and judgement, and patient and family views, opinions and preferences. This approach to decision-making is casuistic in the sense that it acknowledges the uniqueness of the individual (the course of the disease, values, context and the physiological idiosyncrasies) and prevents a pure rule-based method for assigning diagnoses, prognosis and selecting treatment alternatives [1-3]. Nevertheless, in many cases of clinical practice, cancer care is guided by protocols and guidelines, and many clinicians follow algorithms which are rule-based. Rules, guidelines, and algorithms, are useful to the deliberative process, but casuistic judgment is absolutely necessary.

In areas of practice such as children’s cancer care, there is increasing evidence about what works in terms of treatment, but there is still significant uncertainty about prognosis. Childhood cancer illness trajectories are constantly extending resulting in many children outliving original prognostic predictions. This uncertainty means that the decision-making process needs to be casuistic and optimized and adjusted to the situation of each child and their specific circumstances. Children, and young people in particular, present another level of complexity as in most legal systems they are not able to consent until around the age of 16 years (country specific legislation applies) and may or may not agree with their doctors or parents. In these circumstances decision-making, especially when treatment is no longer curative, is particularly complex and highly challenging.

In most scenarios doctors, nurses, parents, children and young people, can come to a shared agreement on a plan of care. Nonetheless, clinicians are aware that parental expectations and beliefs can change over time and are subject to multiple external influences that may be contrary to their own. Clinicians know that managing expectations can be challenging and the decision-making process can become long and drawn out and highly stressful if expectations and beliefs are not shared [3]. Although infrequent, situations can arise whereby children and young people, and or their parents, may not agree with the proposed medical plan and decisions are referred to the High Court for a judgement. By way of illustration, three legal cases are presented in Table 1. In all these cases, the parents declined or withdrew consent to standard treatment for their children with favourable prognosis. In one case, the child asked her mother to halt standard treatment. In all of these cases treatment was ordered over parental objections and over the child’s dissent [4-6]. We found no recent legal cases referred to the Courts in England and Wales to determine care options when treatment was no longer considered curative, which might help with better understanding about the decision-making process. In countries where there is not a medico-legal framework, decisions can sometimes be referred to clinical ethics committees, and where neither exists decision-making can be especially challenging. In this context, ‘law’ and ‘medico-legal framework’ are not confined to a specific jurisprudential framework but instead incorporate international ethical guidelines and declarations, statutory regulations and departmental guidance [7]. Hence, the world ‘law’ also can be thought of as an expression of ethical and moral standards. This spectrum of ‘law’ obviously entails variable impact and outcomes. For example, statutory regulations would be enforceable but they may not be binding on courts.

**Table 1:** Cases in which the parents declined or withdrew standard treatment for their children

**Case and reference**	**Patient age (years)**	**Diagnosis**	**Parent’s desired alternative to standard treatment**	**Child’s desired alternative to standard treatment**	**Court’s decision**	**Patient outcome**
Case 1, Wiener Neustadt (Australia), 1995 [4]	6	Abdominal tumour	To entrust the child’s treatment to a banned German doctor.	____	Mandated standard chemotherapy and surgery.	Completed standard therapy and child was a survivor.
Case 2, Surrey (UK), 2007 [5]	8	Wilms tumour	To halt the last few treatments of radiotherapy as child had had enough.	To halt the last few treatments of radiotherapy	Child Protection Services mandated to complete therapy.	Completed standard therapy and child was a survivor.
Case 3, London (UK) 2013 [6]	7	Medulloblastoma	To prevent her child receiving radiotherapy as she did not believe in its efficacy.	____	Mandated radiotherapy	Child completed standard therapy

### Why is a review needed?

Little is known about the views and experiences of the various actors leading to, or how they came to, a shared agreement when children’s cancer treatment is no longer curative. Scoping of the literature found no relevant published systematic review. The purpose of this review is therefore to explore these issues in greater depth. The review is both timely and important, and evidence is urgently needed to better understand the support that clinicians, children, young people and families need in these challenging and complex situations. Moreover, a high quality theory-informed synthesis of evidence will help policy makers and professionals to determine how to improve end-of-life care of children and their families.

### Conceptual framework

**Figure 1:**
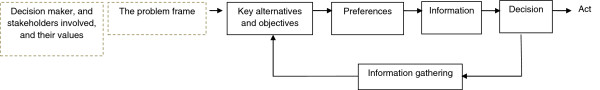
**Howard’s descriptive theoretical decision analysis model**[8]**.**

Howard’s descriptive theoretical decision analysis model [8] originally evolved from statistical decision theory. Howard subsequently laid out a process for solving decision problems and described a decision analysis cycle [8]. Decision analysis is primarily a prescriptive discipline, built on normative and descriptive foundations [1]. The prescriptive perspective focuses on recognizing the limitations and descriptive realities of human judgment. The normative perspective focuses on rational choice and normative models. The descriptive perspective focuses on how real people actually think and behave [1]. Thus, the concepts of Howard’s descriptive theoretical decision analysis model (Figure 1) played an important role in understanding decision-making by different actors in this context. Cognitive psychology (one of the model’s pillars) has a salient position in understanding human behaviour. Decisions are creations of the human mind, and they are manifested in the way that their ‘cognitive structures’ are dynamically self-assembled [1]. A human being (parents, young people, doctors) need data and information, and to understand what it means in order to make a judgement and decision. In this regard, Howard points out three decision essentials: the information one receives; the preferences, and the alternatives. Howard’s model enables focussed exploration of the cognitive processes of decision makers and the decision-making process to develop descriptions of how people actually make judgments and decisions; how a decision is made in light of expectations, values, uncertainties, objectives, and anticipated consequences of each possible choice considered. The model facilitates exploration as to how participants in the decision-making process formulate both the problem and the risk-benefit trade-off of their possible solutions, and to identify factors that facilitate or impede the decision-making process. According to Howard, ‘the problem frame’ is the declaration by the decision maker(s) of what decision is under consideration at this time. The problem frame will influence all elements of the decision basis. The decision maker(s) decide how far away to stand, what to include, and what elements define the figure and the ground. Facilitators and/or barriers to decision-making will also influence all elements of the decision basis.

## Methods

The following objectives were defined to help organise the search and synthesis of evidence:

a. To determine what constitutes best practice within the context of selected medico-legal and ethical guidelines concerning decision-making towards the end-of-life in children and young people with cancer.

b. To explore the evidence about the manner in which the relevant actors make judgments and decisions towards the end-of-life in children and young people with cancer.

c. To identify the factors that facilitate or impede the decision-making process from the perspective of each of the relevant actors.

### Review design

**Figure 2:**
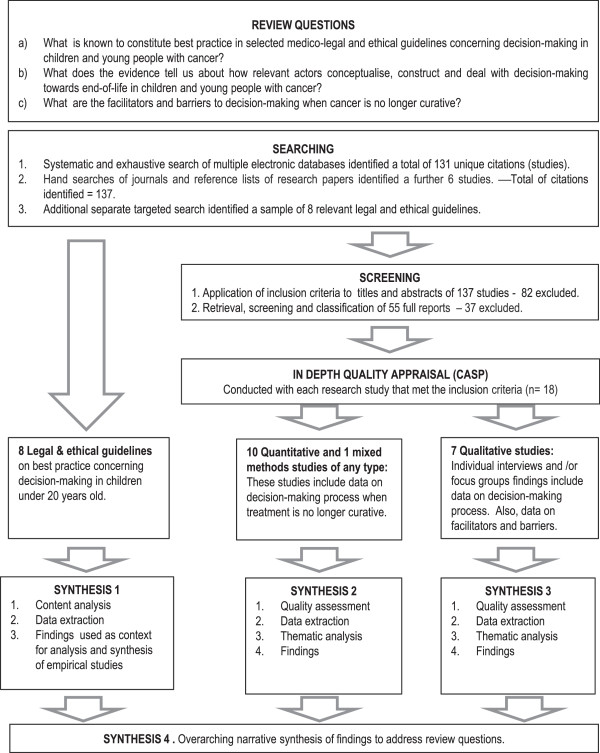
Flow diagram of the review design and processes.

Initial scoping of the literature showed that a mixed-method design was most appropriate to address the objectives. The Evidence for Policy and Practice Information (EPPI) and the EPPI Centre Guidance on synthesis of mixed-method evidence [9] was selected. The EPPI approach was adapted to enable quality screening, and analysis and synthesis of evidence within three separate synthesis streams: ethical guidelines; quantitative and mixed-method studies of any type; and qualitative studies of any type (Figure 2). The analysis of ethical guidelines was designed to address objective a, the synthesis of other quantitative and qualitative studies (streams 2 and 3) was designed to explore objectives b and c. Findings from streams 1 to 3 were then brought together in an overarching synthesis to address the review objectives. Further description of the synthesis processes can be found in the section on data abstraction and synthesis.

### Search strategy

A simple search strategy as advocated by Flemming and Brings [10] was used to locate studies and is summarised in the SPICE table (Table 2) [11] defining the setting, perspective, phenomenon of interest, comparisons, evaluations and methodological approaches. The search strategy was based on key concepts of interest from the objectives. The search terms used included the recognised Medical Subject Heading (MESH). The search terms used included: Minor OR child*; adolescen* OR youth* OR teen*; doctor OR doctor-patient relationship OR clinician; parent* OR family. These keywords were combined with: cancer AND terminal* OR end-of-life OR futil* AND decision-making OR competenc* OR assent AND ethic* OR perception* OR approach* OR experience* OR coping.

**Table 2:** SPICE search strategy

**Setting**	**Perspective**	**Phenomenon ofinterest**	**Comparison**	**Evaluation**	**Methodological approach**
Clinical practice and medico-legal and ethical setting.	Children and young people under 20 years old with cancer, when treatment is no longer curative.	Approaches to and experiences of decision-making. Decision making process when treatment is no longer curative.	Key stakeholders perspectives (children and their parents, and healthcare professionals).	Content analysis of guidelines	Quantitative
					Qualitative
				Comparative and thematic analysis and narrative synthesis of qualitative and mixed method evidence.	Mixed method
					Guidelines
			Ethical guidelines.		

MEDLINE, CINAHL, PsychINFO, Google Scholar, PubMed, Web of Science (Social Science Citation Index), SciELO, The Cochrane Library, Lexis (legal database), Lawtel (legal database), ASSIA (legal database), and Greynet, were searched electronically. In addition, the Web sites of World Health Organization, UNICEF, World Medical Association, European Union, and British Medical Association, were also searched. These searches were focused upon studies published and ethical guidelines launched between 1988 and 2012. These dates were chosen because of the date when the doctrine to respect the autonomy of patients emerged in international legislation. The first seminal publication on the topic of children’s participation in clinical decision-making is the United Nations Convention on the Rights of the Child published in 1989. Yet, even before that, the European charter for children in hospital, 1988, affirmed children’s right to informed participation in decision-making. The electronic searches were supplemented with hand searching of key journals, such as: journal of clinical oncology, paediatrics, and palliative medicine. Inclusion criteria included: (a) Key selected ethical guidelines (limited to a purposive sample from the United Kingdom, Pan European Union, World Medical Association and UNICEF). (b) Quantitative, mixed-method, and qualitative research studies of any type if they reported views, experiences and decision-making by children and/or young people under 19 years with cancer when treatment was no longer curative, and/or by their parents and/or their primary oncologists and/or the clinicians involved. (c) Publications in English and Spanish language. The search for key selected ethical guidelines was purposively limited in order to extract key tenets of international law to serve as context for interpreting evidence from published studies.

The rationale for primarily focusing on decision-making with young people under 19 years reflects typical health service configuration in the UK and Europe, whereby children’s cancer services are typically commissioned for children and young people under 19 years. As previously described, in most legal systems young people are not able to consent until around the age of 16 years (country specific legislation applies), and between age 16 and 18 years parents are frequently involved in supporting their child’s decision-making, especially if their child has lived with a cancer diagnosis for some time. Consequently, regulations and guidelines have sought to protect children and young people in this age range.

Terminology commonly changes from ‘children’ to ‘young people’ around age thirteen, although ‘children’ is commonly used as a term to describe the entire age range. “Youth” is a more fluid category than a fixed age-group. UNESCO uses different definitions of “youth” depending on the context. For activities at international level, UNESCO uses the United Nations universal definition, and defines “youth” as those persons between the ages of 15 and 24 years. For activities at national level, “youth” may be understood in a more flexible manner so we opted to primarily focus on under 19 years in line with health service commissioning by age group, with additional flexibilities to explore perceptions up to age 20 years in studies that primarily mapped onto the under 19 year target age group [12].

### Search outcome

The initial electronic searches identified 131 citations (Figure 2). From these citations, the titles and abstracts were reviewed by EV and checked by JN, of which 55 citations required a full document screen to determine if they met the inclusion criteria. It was unclear whether these studies targeted decision-making processes, paediatric oncologists and other clinicians, children with cancer, and treatment futility. Hand searching identified 6 further research studies that required a full document screen. Eighteen out of 55 studies met the inclusion criteria and were included in this review. Eight guidelines that established criteria to determine which norms should govern ‘best practice’ regarding decision-making towards end-of-life in children and young people with cancer were identified.

### Quality assessment

Studies were appraised within each stream separately using the relevant versions of the Critical Appraisal Skills Programme tool (CASP) [13]. Any disagreements were resolved through discussion between reviewers. None of the 18 included studies were excluded although there were variations in the quality reporting (“Additional file 1”). No study had a fatal flaw (the threshold for exclusion). Guidelines were not appraised critically.

### Data extraction and synthesis

EV extracted and summarized evidence by stream in tables and templates adapted from National Institute for Health and Clinical Excellence (NICE) guidance [14]. Guidelines were subject to content analysis [15] and key guiding principles underpinning ethical decision-making were extracted and summarized in Table 3. Key ethical principles were then used as context to define best practice when interpreting evidence. Quantitative, mixed-method and qualitative streamed and extracted data were summarized in Tables 4 and 5. JN checked data extraction and any queries were resolved by consensus with EV.

**Table 3:** Summary table of international laws, guidelines and regulations upon ethical decision-making in relation to children

**Organization or country**	**Laws (L)**	**Specific provision concerning withholding and withdrawing treatment.**	**Consent requirements**	**Competence**	**Standard for surrogate decision-making**
	**Guideline (G)**				
	**Regulation (R)**				
UNICEF	[G] The United Nations Convention on the Rights of the Child (1989). [16]	___________	Child means a person <18 years of age unless, under the law applicable to the child, majority is attained earlier.	___________	The child’s best interests.
			Article 1.		Articles 3, 18
			The views of the child being given due weight in accordance with the age and maturity of the child. Article 12.		
World Medical Association	[G] Declaration of Ottawa on the right of the child to health care (1998). [17]	To protect every child from unnecessary diagnostic, procedures, treatment…	The wishes of the child being given due weight in accordance with her/his capacity of understanding. Principle 9.	“The mature child, in the judgment of the physician, is entitled to make his/her decisions about healthcare.” Principle 9.	The child’s best interests.
					Principles 4,11
		Principle 4.			
European Union	[G] European Convention on the exercise of Children’s Rights (1996). [18]	___________	Child means a person <18 years of age unless under the law applicable to the child, majority is attained earlier.	It is left to States (the judicial and administrative authority) to define the criteria enabling them to evaluate whether or not children are capable of forming and expressing their own views. Articles 3,6.	The child’s best interests.
European Community					
Council of Europe					
			Article 1.		Articles 1,3.
			Children have the right to express their own views providing they have sufficient competence. Articles 1, 3.		
European association for children in hospital	[G] Charter for children in hospital (1988). [19]	Children shall be protected from unnecessary medical treatment. Article 5.	Children have the right to express their own views providing they have sufficient competence. Article 4.	___________	___________
United Kingdom	[G] British Medical Association 2010. [20]	Futility is the legal and ethical justification for the withdrawal and withholding of treatment. Card 7	All people aged ≥16 are presumed in law to be competent to give their consent to medical treatment. Card 2 In cases of disagreement, the views of the court should be sought. Card 6	The ability to understand that there is a choice and consequences	The child’s best interests. Card 5
				The ability to weigh the information and arrive at a decision.	
				A willingness to make a choice.	
				Card 2	
	[G] General Medical Council. 2010	There is no obligation to give treatment that is futile and burdensome. Legal annex.	Child means a person <18 years of age. Paragraphs 74. Yet they can consent at 16 years. Legal annex.	To assess capacity Paragraph 74.	The child’s best interests. Paragraphs 74,76,77,81.
	GMC’s guidance [21]				
			To involve children and young people in decisions Paragraphs 74,79.		
			In cases of disagreement, the views of the court should be sought. Paragraph 82.		
	[G] Royal College of Paediatrics and Child Health 2004. [22]	Brain Death.	Young people aged over 16 years are presumed in law to be competent to give their consent to medical treatment, but not necessarily dissent decisions. Section 2.4 (2.4.1)	The ability to understand information and to form and express personal views.	The child’s best interests.
		Permanent vegetative state.		Section 2.6(2.6.1)	Section 2.3(2.3.1.2)
			There should be a presumption of competence, unless a child is obviously incompetent. Section 2.6(2.6.1)		
			The wishes and views of the child being given due weight in the light of their		
			knowledge and understanding.		
			Section 2.3(2.3.1.1)		
		The “no chance” situation.	In cases of disagreement, the views of the court should be sought. Section 2.3(2.3.1.2)		
		The “no purpose” situation.			
		The “unbearable” situation.			
United Kingdom	[L_1_] The Children Act (England and Wales) 1989.	___________	Child means a person <16 years of age. [L_2_] Section 2(7).	The ability to understand and to make an informed decision.	The child’s best interests.
	[L_2_] Children Act (Scotland) 1995.		Child ≤12 years of age shall be presumed to be of sufficient age and maturity to form a view”. [L_2_] Section 16(2)		
					[L_1_] Section 1(1)
	[L_3_] The Children Order (Northern Ireland) 1995. [23]				
				[L_1_] Section 43(8) and 44(7)	[L_2_] Section 16(1)
				[L_2_] Section 16(1, 2)	
					[L_3_] Article 3(1)

**Table 4:** Summary table of included quantitative studies

**Reference**	**Objective**	**Study design**	**Participants**	**Setting**	**Results**	**Methods/Quality**
Maurer SH et al. 2010. [24]	Compare the parental self-reported rationale about treatment decisions.	Cross-sectional study.	Parents (n = 62) of children (n = 58) whose disease had progressed to the terminal stage.	1 Hospital: USA	Parental reasons for:	Interview questions pilot tested.
					─Do not resuscitate status or terminal care: concern with quality-of-life (74%) and patient wishes (67%).	Trained interviewers.
		Private and separately semi-structured interviews within 72 hours of participating in non-curative treatment decisions.				
						A convenience sampling technique.
			Most parents were women.			
						No sample size estimation.
					─Phase I Research Controlled Trial: the need to continue cancer-directed treatment (71%).	
					Reasons for both groups of treatment:	Semantic content analysis.
	Treatment decisions:		The children of these parents ranged in age from 0.6 to 21.6 years; median, 11.4 years).			
	─Do not resuscitate or terminal care (47%),					
					To make a decision that did right by their child.	
	─Phase I Research Controlled Trial (53%),					
		Parents were the only decision-makers.				
					To take into account the medical facts.	
					To preserve the child’s legacy.	
Tomlinson D et al. 2011. [25]	To identify the factors influencing decision making about treatment options for end-of-life.	Cross-sectional study.	One parent per child (n = 77; response rate = 67%) of children at end-of-life.	1 Hospital:	Parental factors: hope, increased survival time, and child’s quality-of-life.	Interview questions pilot tested.
				Canada		
						Trained interviewers.
		Single face to face interviews. All items of the questionnaire were closed ended with categoric responses.				
					Healthcare professionals’ factors: financial considerations and parent opinion.	No sample size estimation.
						No probabilistic sampling.
			Median age of children was 8.6 years (range, 6.2-13.3 years).			
						Response rate of healthcare professionals = 100%
	Treatment decisions:					
	─Palliative cytotoxic chemotherapy:					
			Most parents were women (78%).			
	Parents (n = 42, 54.5%),					
						Univariate logistic regression analysis
	Healthcare professionals (n = 20, 15.6%).					
		Hypothetical scenario was presented to parents and to healthcare professionals.				
	─Supportive care alone:					
	Parents (n = 35), Healthcare professionals (n = 108).		Healthcare professionals (n = 128):			
			Primary oncologist (n = 25).			
	p < 0.0001					
			Nurses (n = 97)			
		Factors of options given to them:	Social workers (n = 6).			
		Child’s quality-of-life.	Most healthcare professionals were women (85%).			
		Survival time.				
		Probability of cure.				
Wolfe J et al. 2000 [26]	To describe the primary goal of cancer-directed treatment during end-of-life period.	Cross-sectional study.	One parent per family (n = 103; response rate = 72%) of children who died of cancer (median 3 years; range 1.1-8.0, years after death).	2 Hospitals: USA	Parental goal:	Interview questions pilot tested.
					─To extent life (n = 87, 84%)	
						Trained interviewers.
					Oncologist goal:	
					─To lessen suffering (n = 18, 42%).	
						No sample size estimation.
		All items of the questionnaire were closed ended with categoric responses.				
						No probabilistic sampling.
					(k = 0.16; 95% CI -0.11 - 0.42)	Regression analysis
			Children mean age 10.8 years; SD, 6.7 years old at death.			
		The majority of parental interviews were administered by telephone.				
			Most parents were women (86%).			
			Primary oncologist (n = 42; response rate was not declared). Most of them were men (69%).			
		Only 16% of children participated in the decision-making process.				
De Graves S et al. 2002 [27]	To explore the shift from cure to palliation.	In-depth history audit of medical records.	Medical records of 18 (64%) children who died of cancer. They ranged in age from 2 to 17 years; median age at death 10 years old).	1 Hospital:	For many families the hope of cure continued until the child was close to death.	Pretesting of the audit form used is not reported.
				Australia		
						No probabilistic sampling technique.
					They continued to seek curative or life extending options.	No sample size estimation.
		Parents participated in the decision-making.				
						Content analysis technique.
Bell CJ et al. 2010 [28]	To explore the experiences in adolescents dying from cancer, including end-of-life discussions.	Retrospective review of medical charts.	103 medical charts from adolescents who	1 Hospital: USA	Timing of end-of-life discussions occurred very	Pretesting of the instrument used is
		Parents were directly involved in the end-of-life decision.	died of cancer.		close to death for a significant number of adolescents.	not reported.
						No sample size estimation.
						No probabilistic sampling.
			Children mean age at death 14.4 years; SD, 2.9 years old.			
						Univariable analysis.
Hilden JM et al. 2001 [29]	─ To explore perceived barriers to the delivery of end-of-life care.	A mailed questionnaire survey	228 (55%) paediatric oncologists responded the survey. Gender distribution was not reported.	All members of American Society of Clinical Oncology in the USA, Canada and the UK.	Barriers:	Validated instrument
						Multivariate analysis
					─ Family’s unrealistic expectations for cure (n = 98, 43%).	
					─ Family denial of the illness as terminal (n = 63, 27.6%).	
	─ To describe the factors influencing decision-making.					
					─ Family conflicts (n = 24, 10.5%).	
					Factors influencing d-m:	
					─ Absence of effective therapy (n = 213, 93.4%)	
					─ Request by patient/parent(s) to stop treatment (n = 198, 87%)	
Mack JW et al. 2005 [30]	To identify the determinants of high-quality care at the end-of-life for children, as perceived by parents and physicians.	Cross-sectional survey.	One parent per family (n = 144; response rate = 65%) of children who had died of cancer (a mean of 3.2 years after death).	2 Hospitals: USA	The parents’ principal determinant was doctor-patient communication.	Interview questions pilot tested.
						Trained interviewers.
						No sample size estimation.
		All items of the questionnaire were closed ended with Likert scales.			Physicians’ care ratings depend on biomedical rather than relational aspects of care.	No probabilistic sampling.
						Recall bias.
						Selection bias.
						Multivariable analysis.
			Median age of children at death was 8.9 years (range, 0.3-25.3 years).		No association was found between parent and physician care ratings (p = .88).	
		The majority of interviews were administered by telephone.				
			Most parents were women (83%).			
			Child’s primary oncologist (n = 52; response rate = 100%), most of them were men (65%).			
		All participants were directly involved in the end-of-life decision.				
Edwards KE et al. 2008 [31]	To explore how closely mothers’ and fathers’ understandings of prognosis and treatment goals during the child’s end-of-life period were aligned.	Cross-sectional survey.	Pairs of mothers and fathers (n = 38; response rate = 56%) were interviewed an average of 4 years after their child’s death.	2 Hospitals: USA	During end-of-life, the lessening of suffering was the main primary treatment goal reported. However, only 34% of couples agreed on this goal (k = 0.07; 95% CI, 0.20 to 0.44). During the last month of life, 42% of parents concurred regarding lessening suffering (k = .0.35; 95% CI, 0.05 to 0.65). Among discordant pairs, there was no parental gender preference for a particular goal.	Interview questions pilot tested.
						Trained interviewers unclear.
		The majority of interviews were administered by telephone.				
						No sample size estimation.
						No probabilistic sampling.
						Bivariate analysis.
			Median age of children at death was 10.3 years (range, 0.9-24 years).			
		The items of the questionnaire were closed ended, yet some of them requested open-ended elaboration.				
		All participants were directly involved in the end-of-life decision.				
Hechler T et al. 2008 [32]	To investigate the bereaved parents’ perspective on end-of-life decisions.	Cross-sectional study.	Parents of 48 (Response rate = 35%) children who died of cancer (range 3 - 5, years after death).	6 Hospitals: Germany	64% discussed end-of-life decisions with the healthcare team.	Interview questions pilot tested.
		Semi-structured, single interviews.				Trained interviewers
					Depending on whether parents had had a discussion on decisions with the team, their decision on resuscitation differed.	No sample size estimation.
		The majority of parental interviews were face-to-face.				No probabilistic sampling technique.
			Children mean age 8 years at death, SD, 4.9 years old.			
						Fisher exact test
			Most parents were women (94%).			
	Treatment decisions:	All participants were directly involved in the end-of-life decision.				
	─ Cancer-directed treatment (n = 18)					
	─ Do not resuscitate (n = 24)					
	─ Terminal care (n = 6).					
Mack JW et al. 2008 [33]	To assess parent’s experiences who continued cancer-directed treatment after they had recognized that the child had no realistic chance for cure.	Cross-sectional study.	One parent per family (n = 53) of children who had died of cancer (a mean of 3.2 years after death).	2 Hospitals: USA	The main goals to continue cancer-directed treatment were: cure (20%), life extension (22%) and to lessen suffering (20%).	Interview questions pilot tested.
		All items of the questionnaire were closed ended with categoric responses or Likert scales.				Trained interviewers.
						No sample size estimation.
						Response rate = 64%
					31 parents reported that their child suffered as a result of cancer-directed treatment.	No probabilistic sampling.
			Median age of children at death was 8.9 years (range, 0.3-25.3 years).			
						Multivariable analysis
					29 reported that their child had received little to no benefit.	
		The majority of interviews were administered by telephone.				
			Most parents were women (83%).			
		All participants were directly involved in the end-of-life decision.				
Hinds P et al. 1997 [34]	To identify the factors influencing decision making about treatment options for end-of-life.	Cross-sectional study.	Parents (n = 37, response rate = 44.6%) of children who had died of cancer (6─24 months after death).	1 Hospital: USA	Parental factors:	Interview questions pilot tested.
					Information and recommendations given by healthcare professionals.	Trained interviewers.
		Semi structured interviews were conducted via telephone with parents and face-to face with healthcare professionals. It was then followed up with a questionnaire with a Likert response option.				No probabilistic sampling.
					Oncologist factors:	No sample size estimation.
						Content analysis and
			Children mean age at death 13.4 years; SD, 5.10 years old.		Patient and family preferences.	
	Treatment choices between curative and non curative measures.					Logistic regressions.
					Patient’s prognosis and comorbid conditions.	
			Gender distribution was not reported.		Information and opinions from colleagues.	
			Healthcare professionals			
			(16 oncologists, 3 nurses, and 2 chaplains).			
		All parents were directly involved in the EOL decision.				

**Table 5:** Summary table of included qualitative studies

**Reference**	**Objective**	**Study design**	**Participants**	**Setting**	**Results**	**Methods/Quality**
Hinds Pet al. 200 [35]	To describe the way in which decision are made & factors considered in the decision-making process	Private semi-structured interviews within 24 hours to three weeks of participating in end-of-life decisions.	One parent per family (n = 11) of children whose disease had progressed to the terminal stage.	3 Hospitals: USA, Australia, Hong Kong.	The parental factors identified at all three sites:	Interview schedule piloted.
					“The likely adverse effects of treatment.	Trained interviewers.
						A convenience sample technique.
					“Nothing more to do”.	
					“Believing that my child could not survive”	
						Data saturation not reported.
			The children of these parents ranged in age from 1.8 to 19.11 years).			
	Treatment decisions:				Site-specific factors: the child’s preference, only at the US site (n = 4).	Content analysis technique.
	─ Do not resuscitate status or terminal care (n = 11)					
		All parents assisted in making a treatment-related decision.	Most parents were women.			
					All five Hong Kong parents “felt forced” to participate in the decision-making process.	
Hinds P et al. 2005 [36]	To identify the end-of-life care preferences and the factors that influenced their decisions.	Private and separately, face-to-face semi-structured interviews within 7 days of their participation in end-of-life decisions.	Children (n = 20) aged 10 to 20 years; mean, 17.4 years.	2 Hospitals: USA, Australia.	Children factors:	Interview schedule piloted.
					Caring about others	
					Avoiding adverse effects.	Trained interviewers.
					Parental factors:	A convenience sample technique.
			Parents (n = 19)		The child preferences.	
					Trying for cure	
						Data saturation not reported.
						Semantic content analysis.
			Oncologist (n = 14), Most parents and children were women.		Oncologist factors: Patient’s prognosis and comorbid conditions.	
	Treatment decisions:					
			All participants were directly involved in the end-of-life decision.			
					Patient and family preferences.	
	─DNR status (n = 5),					
	─Terminal care (n = 7),					
	─Phase I RCT (n = 8).					
Hinds P et al. 2009 [37]	To identify parental definitions of being a good parent. And the actions from clinicians that would be helpful to them in fulfilling this role.	Private and separately, face-to-face, semi-structured interviews within 72 hours of participating in no curative treatment decisions.	Parents (n = 62) of children (n = 58) whose disease had progressed to the terminal stage.	1 Hospital: USA	Good parent means: (i) Doing right by my child. (ii) Making decisions in the child’s best interest. (ii) Meeting the child’s basic needs.	Interview schedule piloted.
						Trained interviewers.
						Convenience sampling technique.
			The children of these parents ranged in age from 0.6 to 21.6 years; median, 11.4 years).		Actions from clinicians:	Data saturation not reported.
					To know that the child was receiving the best clinical care.	
	Treatment decisions:					Semantic content analysis.
	─Do not resuscitate status or terminal care (48.3%),				Every theme was reflected in all three decision types.	
			Most parents were women.			
			Parents participated in the decision-making.			
	─Phase I Research Controlled Trial (51.7%),					
Tomlinson D et al. 2006 [38]	To identify the factors influencing decision making about treatment options for end-of-life.	One focus group.	Parents (n = 7) of children (n = 5) who had died of cancer (from 0.6 to 14 years after death).	1 Hospital: Canada	Parental factors for chemotherapy:	Interview schedule piloted.
						Trained interviewers.
					Hope, time, relieve pain, child’s decision.	
						A convenience sampling technique.
		Hypothetical situation was presented to parents.				
					For supportive care:	
						Data saturation not reported.
			Age of children at death no declared.		Time, lessening suffering, nothing more to do, child preferences.	Content analysis.
		Factors options:	Most parents were women.			
	Treatment offered:					
			Parents were the decision-makers.			
		Child’s quality-of-life, Survival time, Probability of cure.				
	─Palliative cytotoxic chemotherapy;					
	─Supportive care alone.					
	Percentages per group no declared.					
Hannan J et al. 2005 [39]	To identify the factors influencing decision making about final place of care (home or hospital).	Private and separately open-ended interviews. Parents were the only decision-makers.	Parents of children (n = 5) who had died of cancer (from 1 to 2 years after death).	1Hospital: England	Valuing time left,	Interview schedule piloted.
					Needing to feel safe and secure, and	Trained interviewer.
					We did not know what to expect.	A purposive sampling technique.
					No difference between home and hospital, other than the desire to have control themselves as a family.	Data saturation not reported.
			Gender distribution was not reported.			
						Interpretative phenomenological analysis.
			The children of these parents ranged in age from 10 to 19 years).			
	Place of care decisions:					
	Home (n = 3)					
	Hospital (n = 2)					
Bluebond LM et al. 2007 [40]	Parents’ approaches to care and treatment.	Ethnographic study including	Parents of 34 children whose disease had progressed to the terminal stage.	2 Hospitals:	23 accepted CDT or look on their own or asked their doctor to do so.	Interview schedule piloted —unclear.
				USA		Trained interviewer.
					4 out of 23 cases to whom was offered CDT declined CDT.	
						Convenience sampling technique.
				UK		
		Participant observation, Open-ended, semi structured interviews. Parents were the only decision-makers.			2 out of 11 cases to whom was offered only TC agreed.	Data saturation not reported.
			The children ranged in age from 0.9 to 19.7 years; median 6.0 years).			
						Constant comparison analysis technique.
	Treatment offered:					
			Gender distribution was not declared.			
	Cancer-directed treatment (n = 23), Terminal care (n = 11),					
Steward JL et al. 2012 [41]	To describe and explicate the treatment decision-making process from the perspectives of parents.	Private and separately semi structured interviews, within six months of participating in major treatment decision-making.	Parents (n = 15) of children (n = 13) whose disease had progressed to the terminal stage.	3 Hospitals: USA	Parental motivations for making the right decision:	Interview schedule piloted.
					(i) Doing right by my child. (ii) Making decisions in the child’s best interest. (ii) Meeting the child’s basic needs.	Trained interviewers.
			The children of these parents ranged in age from 3 to 17 years; median, 10 years.			
			Most parents were women.			Convenience sampling technique.
			Parents participated in the decision-making.			
						Data saturation not reported.
	Treatment decisions:					
	─ Hematopoietic cell transplantation (n = 5),					Constant comparative analytic process.
	─ Research Controlled Trial (n = 8).					

It was not possible to perform a meta-analysis as we did not find any clinical trials and observational studies were heterogeneous and did not permit statistical pooling. Therefore, a thematic synthesis approach [42] was used to synthesise evidence from streams 2 and 3. We undertook a thematic synthesis with 18 studies (by stream: 10 quantitative studies, 1 mixed method study and 7 qualitative studies). It is important to note that the process of coding primary studies was initially undertaken in an inductive way. Once data were coded and organized thematically, we drew on Howard’s descriptive theoretical decision analysis model (Figure 1) as a framework for understanding decision-making. We used Howard’s model to focus on the decision process, including factors that were considered and the anticipated outcomes, rather than on assessing decision quality (correctness or appropriateness) [1,8]. We identified decision-makers and stakeholders involved in the problem, unpicked their values and their preferences to be used for the decision, and identified key alternatives and objectives and information available.

The analysis process required each study to be read repeatedly to ensure that all concepts were integrated and the relationships between the concepts of each study were explored. We used the notion of first order, second order and third order constructs to analyze and reinterpret the studies [42]. First order constructs are insights offered by participants in the original study. All participant quotations that were paraphrased by the original researchers were extracted as first order constructs. Second-order constructs are the interpretative themes that were developed by the original researchers from the first order constructs. We described and listed the themes reported by authors of each original study and made a note of the number of studies that contributed to each theme. Third order constructs were derived from the synthesis of evidence across multiple studies. We developed third-order constructs by analyzing the second-order constructs to identify new, common themes that emerged from our inductive analysis to address the review objectives. Data entry and analysis were performed using Atlas/ti version 7 computer software.

### Interpreting the entire dataset and developing a new theoretical framework

**Figure 3:**
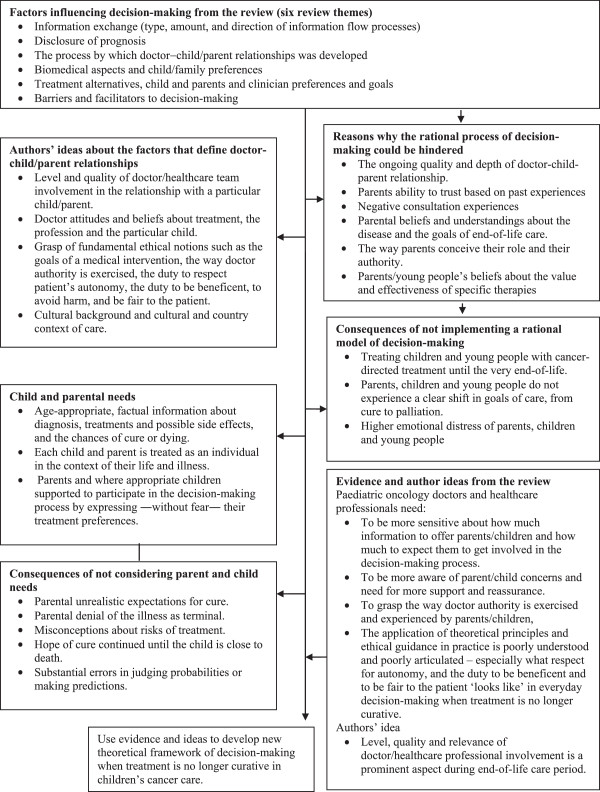
**Factors influencing decision-making and ideas and hypothesis generation from relevant actor and author perspectives ―when treatment is no longer curative.** Level 3 constructs.

**Figure 4:**
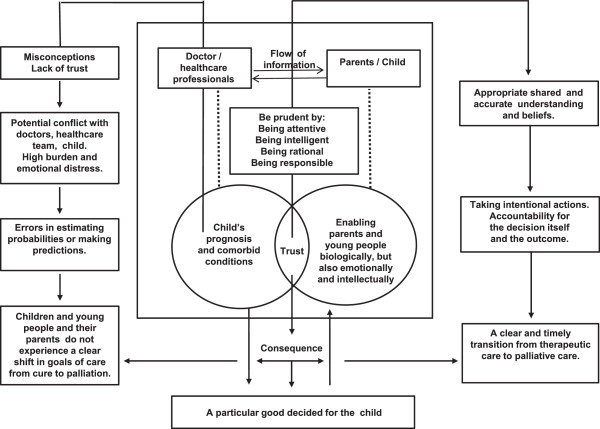
Theoretical framework of the positive and negative influences in the decision-making process.

Finally, we synthesised evidence from the three streams (Figure 2) in an overarching fourth narrative synthesis. We juxtaposed key tenets of best ethical practice in key selected guidelines (such as ‘best interests’, consent requirements, age to assent/consent, etc. described in Table 3) against the themes from 18 studies using a constant comparison technique [15] in order to see the extent to which these tenets are legally or socially enforced. First level constructs (participant quotes) and second level constructs (primary research author interpretations) were translated into the 6 themes (third order constructs) to which we then added our own ideas and interpretations through ongoing engagement with the evidence and using our expert knowledge of the field to form hypotheses concerning what the evidence said about factors influencing decision-making from relevant actors’ perspectives (Figure 3). During several subsequent meetings, the authors used Howard’s model to further inform organisation and interpretation of ideas to help them understand how the relevant actors of the doctor-patient-relationship shaped the goals, objectives and preferences in treatment decision-making when cancer treatment is no longer curative, and over time developed a new theoretical framework to help caregivers to identify the changes required of them and for guiding future research (Figure 4).

## Results

### Key principles and tenets from ethical guidelines (stream 1)

Eight guidelines about decision-making towards the end-of-life, in children and young people with cancer, were purposively sampled, reviewed and then summarized [16-23] (Table 3) in order to determine what constitutes best practice in accordance with commonly accepted medico-legal and ethical guidelines. The guidelines selected include the most comprehensive documents on the substantive rights and obligations to protect the child patient’s vulnerability. Many medical societies worldwide, including the British Medical Association, are bound to them by international law.

#### What constitutes best practice in legal and ethical guidelines?

Children age 16 to 18 years are presumed to be competent to give consent to medical treatment as if they were adult (age 18 years in the UK). However, if both the parents and their child refuse treatment, the court can override the refusal if it is in the best interest of the child to do so [4-6]. Children under 16 lack ordinary legal authority to make binding medical decisions, yet they could in specific circumstances meet functional criteria and be declared competent to do so. In such situations legal arguments support giving decisional authority to the minor patient [16-18,20-23]. In this group of children, the best interest of the child is always a primary consideration (Table 3). Whilst the guidelines outline consistent ethical principles, there is however an absence of guidance on the application to real life clinical scenarios.

### Actor perspectives and decision-making towards the end-of-life (streams 2 and 3)

Quantitative studies on actor perspectives included cross-sectional structured interviews with parents [24-26,30-34] and with paediatric oncologists [25,26,34], a mailed survey with paediatric oncologists [29], one in depth history audit of medical records [27]; and a review of medical charts [28]. Most of the studies [26,30-34] included bereaved parents 0.6 to 5 years after their child’s death.

Qualitative studies included semi-structured interviews with parents [35-39,41] and with parents/children/oncologists [36], one ethnographic study [40], and one focus group [38]. Only three studies [34,38,39] included bereaved parents with 1 to 3 years after their child’s death.

Synthesis of 18 included studies identified six overarching themes: (i) the flow of information to inform decision-making; (ii) disclosure of prognosis; (iii) the process by which doctor-child/parent relationships were developed; (iv) biomedical aspects and child/family preferences; (v) alternatives of treatment, preferences, and objectives and goals of their preferences, and (vi) barriers and facilitators to decision-making.

#### Information exchange (type, amount, and direction of information flow processes)

All parents recognized that they had assisted in making a treatment-related decision on behalf on their child [24,27,32,35-41]. Hinds [37] and Steward [41] reported that all US parents willingly assumed the burden because they considered that it was their job and they never considered shirking this critical responsibility. However, in a cross-cultural study (US, Australia and Hong Kong) Hinds [35] found that all five Hong Kong parents were “feeling forced” to participate in the decision-making process, thereby showing that cultural context is an important consideration.

The type and amount of information exchanged between clinicians and parents, and whether information flow was really two ways, was not reported. However, nearly all parents described an interactive process between themselves and the healthcare team, mostly with their child’s primary oncologist [26-28,32,35,36,40,41]. Some parents reported that their child’s primary nurse, or the psychosocial clinician [26,32], or another doctor [28], were involved at some point in the discussions about whether their child had no realistic chance of being cured. Only few parents denied having discussed this with the clinical team [28,32]. In a few of these cases, it was reported that a conflict existed involving disagreement between clinicians and parent(s), with the parent(s) not yet ready to agree to a change in ‘do not resuscitate’ status [28].

Some studies [26,28,30,36,40,41] clearly identified whether or not the children and young people were included in the discussion regarding treatment. There was variability in the degree to which parents elected to involve their child in the decision-making process. Mack [30] and Steward [41] reported that some parents deliberately excluded their child, either because they felt their child was too young to participate or to spare even older children from the burden of participating in the decision. Among children who were not perceived (by their parents) to be too young (median age 10 years, range 3—17 years), the majority received information via direct communication from the doctor [26,30,36,41]. In contrast, according to the US parents interviewed, the child’s preference was of high importance in end-of-life care decision-making irrespective of the child’s age [24,26,35,36,38,39,41]. The children’s ages of this group of parents ranged from 0.6 to 21.6 years, with a median of 13.5 years. The studies did not organise findings by children’s age to shed more light on age-related decision-making processes and assent and consent issues. Only one study [36] reported to have interviewed directly a sample of children to identify the factors that influenced their end-of-life care decisions, the child participants in this study were aged 10-20 years.

#### Disclosure of prognosis

For parents and young people, disclosure of prognosis and the process by which doctor-child/parent relationships were developed were important when making judgements and decisions towards the end-of-life in children and young people with cancer. For oncologists, biomedical aspects (child prognosis and comorbid conditions) and child/family preferences regarding the options that respond to the problem frame figured most importantly in their decision-making.

Terminal care was defined as cessation of cancer-directed treatment, which may or may not continue with aggressive symptom management [24,36,37]. The term palliative care was used to make reference to the initiation of palliative measures when there was no longer a reasonable hope of cure, or to refer to supportive care [27,38,40]. The terminal stage was reached when, according to child’s primary oncologist, standard therapy had failed and cure was no longer a possibility [24-28,30,31,37,38,40]. The terminal stage involved the prediction and evaluation of outcomes, which were usually probable rather than certain. The trajectory to death of those who had reached this stage was lower than or slightly longer than 3 months [26,28,31,38,40], or in one study less than 6 months [32]. Some clinicians initiated disclosures when cancer had recurred and it had less than 30% chance of cure [25,38,40]. End-of-life period was defined as the time before death and it was related to incurable cancer and its terminal stage. End-of-life discussions more likely occurred in the last 7 days of life, [28] or late in treatment [34].

Parents and mostly proxy reports of children’s views showed that irrespective of context they wanted their doctors to appear interested, listen well, explain clearly, be open to discussion and achieve verbal agreement with them [24,27,30,32,34-38,40]. Time to allow other possibilities like a second opinion and/or alternative therapies [27,35,40] were important during the end-of-life period. Other factors impacted on the effectiveness of end-of-life communication, such as the need for hopeful messages about a possibility for cure, or longer life expectancy, or related to short term visions of the future, or continued care, or an indication that the clinician had not given up (children) and, that they had not given up on their child (parents) [24-28,31,36-38,40]. The single study conducted with children and young people [36] showed that they preferred to be informed even when the prognosis was poor or treatment was no longer curative.

#### The process by which doctor-child/parent relationships was developed

Trust was the single most important factor in the decision-making process and there was variation in trust afforded by parents to doctors. Trust was in part a product of clinician behaviour. Several parents looked for medical facts that included the trustworthy doctor’s expertise (clear and understandable information about their child’s condition) or medical knowledge obtained through research [24,30,32,34-38,41]. Many parents reported a need to believe that clinicians were giving to their child the best possible clinical care and that they (parents) could count on clinicians to guide the decision-making [34-38,40,41].

A few parents acknowledged that they really had no choice but to trust in individual caregivers and in the profession of medicine (paediatric oncology specifically) given their child’s situation [41]. For these parents, trusting doctors and medical knowledge eased the pressure they felt to make the right decision [41]. Only one parent articulated a lack of trust in her child’s clinical team, specifically voicing scepticism about the doctor’s motives for presenting a clinical trial in a positive light in order to enrol her child. The scepticism towards the motives of the doctor intensified the difficulty of the decision-making process for parents [41].

Whilst the evidence suggests that variation in trust is associated with clinician behaviour, the variation may be highly individual and reflect a parent’s own health beliefs concerning the appropriateness of experimental or ‘rescue’ therapies or their individual ability to trust others that predates the child’s illness. The same clinician behaviour that is perceived as collaborative and in the best interest of their child by one parent may be perceived as intrusive, threatening or experimental treatment by another. Parents also stressed the importance of needing to be treated as individuals. Parental assessments of doctor attributes rested, largely, on their perceptions of the doctor’s ability and willingness to contextualize the decision-making process by framing the end-of-life discussion in terms of each child’s unique background, characteristics and life experience [24,35,36,41]. From the parent’s perspective the expectation was always that the doctor would facilitate this process. Both groups (parents and children) [24] desired that staff should continue to provide thoughtful care, including emotional care. Parents cited actions, for example: honesty, caring, sensitivity, and treating each child uniquely [30,37,38]. There was a noticeable lack of evidence concerning the wider complexities of decision-making and the complex beliefs and experiences of parents that could help or hinder the process of developing trusting relationships with clinicians.

#### Biomedical aspects and child/family preferences

To all oncologists, the child’s prognosis and comorbid conditions were among the central considerations of their decision-making [25,26,29,34,36]. This evidenced their desire to avoid harm and to lessen suffering in the absence of effective therapy. A survey of 52 paediatric oncologists and 144 parents showed that the highest doctors’ ratings of quality of end-of-life care were characterized by ‘little pain’ and ‘minimal time in the hospital in the last month of life’, whereas parents’ highest ratings were ‘clear information, given in a sensitive caring manner, about what to expect in the end-of-life-period’ [30]. No association was found between parent and doctor ratings of quality of end-of-life care [30]. All participating doctors also marked child/family preferences as important [25,26,29,34,36].

#### Treatment alternatives, child and family and clinician preferences and goals

Parents and children typically faced one of two alternative decisions: doing something or doing nothing. The first was a choice of cancer-directed treatment, randomized or non-randomized controlled trial, or terminal care (end-of-life palliative care) with or without palliative chemotherapy. The second included the choice of withdrawal or withholding treatment and the decision to not resuscitate. The selection of an option was based on the decision-maker’s goals and objectives.

#### Cancer-directed treatment

Parents with inaccurate perceptions of the prognosis or unrealistic expectations for cure were more likely to choose aggressive therapies, including cancer-directed treatment to overcome disease and promote recovery, than parents with more realistic expectations. The decision for cancer-directed treatment in the end-of-life care period occurred more frequently in those parents who had not had a discussion with the clinical team on the topic beforehand [27,32] or when trust was not apparent or shared in the decision-making process [27]. There were also parents who recognized that cure was unlikely, and even then they elected to continue cancer curative treatment. These parents pursued different goals, such as: to keep hoping [26,27,33,40]; to ensure that everything had been done [26,33]; to have more time with their child [40]; or life expectancy extension [26,33]. Few parents reported that the primary goal of cancer-directed treatment during this period was to lessen suffering [26,31]. In contrast, oncologists in surveys mentioned ‘the family choices’ as a primary goal [26,34].

#### Recruitment to clinical trials

Parents saw their primary goal as advocating for their child the best chance for a cure [24,36,41]. Deciding to enrol their child in a trial of experimental treatment was commonly interpreted by parents as being offered a chance for cure, albeit sometimes a very slim or even highly unlikely chance of cure depending on the type of trial. Within this decision-making scenario parents also weighed up and considered secondary goals such as preserving the child’s present and future quality of life [41]. Another parental goal included the desire to help others by means of cancer research [33,36,41]. Young people reported that for them, their goals were to extend their life expectancy ―specifically to cure, and to want to help others by means of cancer research [36]. In the same study, doctors declared that their goal was wanting to benefit their patients and others, thereby indicating a dual purpose in wanting to do the best for individual children, but also to use their knowledge and clinical experience of treating individuals, to advance the care and treatment for children with cancer generally (*ie* a greater good) [36].

#### Terminal care

All parents that chose terminal care without palliative chemotherapy (supportive care alone) voiced many reasons, but the most common goal was to pursue their child’s quality of life, by diminishing the suffering of their child [24,34-36,38]. The following considerations were influential factors in their decision-making: valuing time left [36,38], nothing more left to do [24,35,36,38], believing that nothing else could really help the child [34], thinking the child would never get better [34]. The primary goal for those parents who chose terminal care with palliative chemotherapy was also their child’s quality of life, and as a secondary goal to create a sense of hope for a cure and an extension of life expectancy [25,38]. The decision to opt for terminal care in the end-of-life period occurred more frequently in those parents who had held a discussion with the clinical team on the topic [24,32,34,35]. The goals typically mentioned by young people were also focused on their quality of their life, the avoidance of cancer-directed treatment adverse effects, the belief that further treatment is futile, the feeling of being ready to die or the experience of having seen someone else die [36]. Oncologists saw as their primary goal to avoid harm when there was no other option or the absence of effective therapy [29,34,36].

#### Withdrawal, withholding, and do not resuscitate

Young people, parents and clinicians pursued the same terminal care goals, that is to improve life conditions with good symptom control and quality of death in this group of children [24,32,34-37]. A few families considered the financial cost of further treatment for their child as a factor that facilitated their decision to forgo any further active treatment [27].

### Place of death

Most children and young people died from disease progression in a hospital setting [27,28,31,32,39]. The parents’ goal was to be in control at the time their child’s death [39]. More than a third of 228 paediatric oncologists surveyed in the US, Canada and UK stated that the lack of a readily available and easy-to-use palliative care team or pain service made the delivery of good terminal care difficult [29].

#### Barriers and facilitators to decision-making

One study identified the barriers and facilitators to end-of-life care [29]. Five studies identified the actions from staff that helped or did not help in the decision-making process [24,34-36,38].

The barriers identified most often according to parents included: the fondness that staff members showed their child that made it more difficult (for parents) to make a decision to stop cancer-directed treatment [35], feeling forced to decide from alternative care pathway options [35], not having written information about the drug in a trial [36], and financial burden [38]. For young people, the barriers related to the possible toxicities of the experimental drug being tested in a trial [36]. In contrast, for oncologists, the barriers were commonly the family having unrealistic expectations for cure [29], family denial of the illness as terminal [29], and internal family conflicts [29].

The facilitators most often identified by parents were: the provision of thoughtful care by staff [24,34-36,38], trusting the staff [24,34,35], getting clear information from the clinical team throughout the entire process of decision-making [34-36], and the support to them/their child from the clinical team [24,34,36]. For young people, facilitators included: the provision of thoughtful care by staff [36], getting clear information from the clinical team [36], and receiving support from the clinical team [36]. For oncologists, facilitators included: staff agreement on option chosen [34,36], and ability of the child and family to understand and accept the situation [34,36].

### Overarching narrative synthesis of the entire dataset and development of a theoretical framework

The overarching narrative synthesis focused on moving beyond the thematic analysis to mapping ideas and generating and interrogating relationships in the synthesised body of evidence with Howard’s model in order to develop a theoretical framework of the positive and negative influences on the decision-making process when the treatment is no longer curative. The process began with privileging factors influencing decision-making from the perspective of the relevant actors as outlined in Figure 3, which were then used as a basis for developing a theoretical framework (Figure 4). The logic of the theoretical framework (Figure 4) is described in the following paragraphs.

Evidence suggests that when doctors believe that their work is to cure disease or maximize medical outcomes, the doctor’s clinical role in decision-making is restricted to giving the parents/children all relevant research evidence on the benefits and risks of various treatment options so that they will be able to make an informed decision alone. In the Figure 4 this notion is depicted in the left-hand circle, at the centre of the figure. Child prognosis and comorbid conditions - when treatment is no longer curative - were the predominant motivations in the doctors’ decision. The right-hand circle represents, theoretically, the subjective realm of health and illness. Both circles ought to be inextricably joined. Yet, the evidence showed that doctors did not generally dig deeper to explore wider life-course determinants that may impact on a parent’s ability to trust them. This superficial level of doctor involvement in the relationship with the children’s parents resulted in either lack of parental trust, or meant that parents made substantial errors when judging their child’s prognosis. Hence, for many parents, participating in decision-making represented a high burden and emotional distress, conflict with the clinical team, and the constant searching for cancer directed treatments. This resulted in children and young people who did not experience a clear and timely shift in the goals of care, from curative to palliative, over time.

Whereas, when doctors established a collaborative relationship with the child and their parents, and showed loyalty to what parents and their children were demanding of them (to be attentive to all the evidence, understand the evidence intelligently, rationally judge whether their understanding was correct, and act at the right time based on what has been understood correctly), parents felt understood and treated as persons. They trusted their doctors and clinical team, thereby decreasing the emotional distress, and their children experienced a timely and clear transition from therapeutic care to palliation.

Whilst showing a trajectory of positive and negative outcomes, it is likely that families will move along a continuum depending on the decision, the illness trajectory and the varying perspectives of different clinicians that they encounter. Parents’ role as protectors and their desire to prevent harm in combination with the variety of treatment options, outcomes and prognosis makes decision-making very hard and some are likely to waver or alter their decisions over time.

## Discussion

A child’s age-appropriate competence to fully understand and to express personal views, and the ‘best interest’ standard, is prescribed by the medico-legal and ethical guidelines and generally followed during decision-making processes in actual clinical practice. The term competence for assent differs from the term competence for consent (a legally valid authorization). Thus, according to the medico-legal and ethical guidelines, age has conventionally been used as an operational criterion of valid authorization. Thresholds of age (for children under 16 years-old) vary in accordance with a community’s standards, with the degree of risk involved and with the importance of the prospective benefits.

Application of these two criteria of best practice (age-related competence and mental capacity and best-interest concerning decision-making towards end-of-life in children and young people with cancer) help parents and doctors arrive at what is considered by them as the best decision for each child. Nonetheless, what constitutes the ‘best interest’ course of action from various actor perspectives varied considerably.

To clinicians, oncologists in particular, the measures of success or failure of treatment were predominantly quantifiable, emphasising severity of disease and life expectancy. Moreover, the value of ‘respect for autonomy’ was understood as having to follow the ‘child/parents’ preferences’ limiting their role to information transfer (*i.e.,* to give relevant information about the patient’s prognosis, and benefits and risks of various treatments so that parents were enabled to make an informed decision). Doctors also understood that the remaining task of deliberation and decision-making was generally the responsibility of the parents/family alone, unless the child was legally competent to make the decision. So, within this decision frame, acting in the child’s best interest is a medical/biological choice rather than a moral choice. Hence, from the doctors’ perspective, unrealistic parental expectations for cure were considered to be the biggest barrier that impacted on implementation of best-practice guidance. Beauchamp and Childress [43] however consider that neither the children, their parents nor their clinicians have premier and overriding authority, and no pre-eminent principle exists in medical ethics. Beneficence provides the primary goal and rationale of medicine and clinical practice, whereas respect for autonomy, along with nonmaleficence and justice, sets moral limits on the clinician’s actions in pursuit of this goal. To achieve this, interpersonal relationships and the core issue of trust between child-parent-clinicians should and ought to be given a co-execution of the acts in which and with which child, parents, and clinicians, execute their status as moral agents [43]. Nonetheless, there was a noticeable lack of evidence concerning the wider complexities of decision-making and the complex beliefs and experiences of parents that could help or hinder the process of developing trusting relationships with clinicians. The ability of researchers to better understand these complexities is likely to depend on the availability of additional evidence that provides a greater degree of insight into these complexities from multiple informant perspectives using longitudinal ethnographic methods.

Similar to the present discussion of ‘best-interests standard’, the literature on this topic has previously distinguished between best-interests standard as an absolute duty or rule, and best-interests standard as a very general guideline; thereby pointing to the negative effects of a deontological approach in which ethics consists of binding rule-based obligations that responsible actors can be expected to know and put into practice [44]. The findings of this review show that although doctor concern, morally speaking, has to do what was the best for the child, the basis of their actions was merely deontological or rule based by always acting according to a specific view of what is ‘right’.

To parents, increasing the length of life expectancy and curing their child’s condition remained as important goals but were not always as important as the child’s quality of life and suffering. It was clear that, parental treatment preferences were rooted in the quality of the interpersonal process that occurred during consultations and in the level and depth of the oncologists and other clinicians’ involvement. Within the parental decision frame, parents faced a difficult question about whether to emphasize respecting their child’s autonomy or to protect their child from harm –a moral rather than a medical choice. This problem becomes, for them, more difficult to solve when doctors fail to recognise the complexity and changeable nature of the desires, emotions, and needs that characterise the doctor-child-parent relationship, especially during end-of-life care.

To young people, quality of life and avoiding adverse effects was the main goal for making judgments and decisions. Concern and caring about others was the most influential factor in their treatment preferences Furthermore, similar to their parents, their treatment preferences were rooted in the perceived quality of the interpersonal process that occurs during consultations and in the level and depth of the oncologists and other clinician involvement. So, within the young persons decision frame, their decision-making was moral based.

A child’s age-related competence to understand and decide is considered as a pivotal factor for valid consent. So, to have the ability to consent to, or refuse, treatment means that children must be legally competent. There exist different tests to indicate whether the child has ‘sufficient understanding and intelligence to enable him or her to understand fully’. Nonetheless, all methods for setting standards of competence are defined in theory-oriented guidelines, rather than operationalised by practice oriented professionals [43,45,46]. With regard to this issue, it was noted that findings from children did not feature strongly. Nevertheless, the empirical evidence shows that emotional and moral aspects, such as children and young people’s expectations about their role in choice and decision-making, and the effects of the child’s decisional authority on treatment decisions were not considered in the studies reviewed.

The range of decision-making issues identified point to the need to improve understanding of decision-making processes in this specific context for children and young people. Ridd et al. [47] propose three critical elements of the doctor-patient relationship (continuity of care over time, positive consultation experiences, and depth/involvement of relationship) as indicators of the quality of the care of adult patients. Our findings suggest that the experience of children and young people and their parents, with their clinical providers, constituted a powerful and influential factor in the development of trust in the doctor-child-parent relationship. The level of doctor involvement impacted in the same way. Trust also appeared as the most important ingredient for parents. Lack of trust in the clinicians or belief in the plan of care offered were the primary reasons for choosing to continue cancer-directed treatment. Additionally, there were differences in importance between the values of parents and oncologists. Parents held values such as honesty, caring, sensitivity, thoughtfulness, etc. highly, and they identified them as influencing their treatment preferences. In contrast, the values held by the child’s primary oncologist had to do with the child’s prognosis and comorbid conditions. From the values held, it may be said that the aim sought by parents was maximizing their understanding about prognosis, and the aim of doctors was maximizing medical outcomes. These findings confirm what some clinicians have said about decision-making, that values, feelings, skills, and personal background, make evident why one thinks and acts as one does [1,48]. Therefore, it is strongly recommended that a deeper level of doctor (and wider clinical team) involvement should include asking about feelings and acknowledging and legitimizing emotions, to assist in exploring the thoughts that parents/children have about hopes for their future.

### Limitations

The majority of empirical research about the decision-making process when cancer treatment is no longer curative, in children and young people, is based primarily upon interviews with bereaved parents (≤5 years after their children died), and to a lesser extent, interviews with children’s primary oncologists, and medical record reviews. Most parents (n = 530/629, 84%), young people (n = 14/20, 70%), and clinicians (n = 142/236, 60%) were female. Only one study [36] included the words of the child. All studies were carried out in high income countries (US, Canada, UK, Germany, Australia, and Hong Kong) and with English speakers. Given the centrality of values, beliefs and patterns of communication during the treatment decision-making process, it is possible that a different picture could have emerged from a population sample that represented middle-income and developing countries.

There were also some important knowledge gaps, and gaps in understanding in the available evidence. Although the voice of the child and the child’s increasing autonomy over time as they grow up is an important consideration, evidence did not shed much light on developmental aspects of decision-making, nor did children and young people with cognitive impairments feature in included studies. This issue is relevant as many children and young people gain mental capacity to make decisions as they grow up but can lose capacity or ability to engage with decision-making as their condition worsens. Many children and young people experience critical lapses in their condition which would mean their parents having to act as sole decision-makers working with their clinicians either permanently or until a point is reached when the child recovers sufficiently to re-engage in decision-making on some level.

Clinician perspectives are currently more likely to be captured in large quantitative surveys than in high quality and rich ethnographic qualitative research. Quantitative surveys are more likely to reflect the values of the researchers than the researched. Also worthy of mention is the notion that a reliance on key tenets of ethical practice may over privilege ‘top down’ guidelines as a source of moral authority. Counteracting this notion of ‘over privileging’ is the process by which many of the guidelines were developed by consensus and subjected to extensive public consultation. In addition, by including observations from those close to the realities and complexities of real life clinical practice, additional valuable insights into the clinical application of guidelines are incorporated into the synthesis.

Although the low number of medico-legal cases indicate that fundamental differences in opinion between actors are rare, it is likely that parents and their children will not always agree on aspects of decisions or specific choices, options and decisions for some or all of the time. Likewise, biological parents may not hold the same ideas and aspirations for their child’s care and treatment, and decision-making may be complicated further if step-parents and reconstituted family structures are involved. Parents and children, either individually or collectively, may change their minds about the best course of action over time. Similarly, parents used to making decisions for their young children may experience difficulties having to accommodate their growing children in the decision-making process as their child’s autonomy and understanding increases. Nonetheless, the evidence is not sufficiently nuanced to draw out different actor perspectives, decision-making contexts, or to differentiate between subtle or marked differences in opinion, nor the processes by which these differences may or may not be overcome in the decision-making process. There is also far more evidence from parents and the child’s perspective is critically under researched and under represented. The single study that did however include children and young people’s views and experiences provided a snapshot of how they were positioned in the decision-making process at different ages, and some important insights such as the altruism of some children wanting others to benefit from their experience.

There is a wealth of evidence from studies undertaken with children and young people with non-cancer life-limiting conditions indicating there are major challenges in the way clinicians communicate, exchange information and involve children and young people in decision-making processes [49]. There is as yet insufficient evidence to show whether these same challenges exist in situations when cancer care is no longer curative. In developing the theoretical model there was insufficient evidence to locate the child or even the young person age 16-18 years as potentially having a spectrum of different age and developmentally-appropriate views, opinions and experiences from their parents, and there was insufficient evidence to differentiate parental views or reconstituted family contexts whereby the child may have separated or additional step parents who may contribute to the decision-making process. Wider family perspectives (such as siblings) are also largely absent.

The fact that evidence mainly comes from parents and doctors, indicates that the complexity of decision making by relevant actors is not well understood as a non-linear longitudinal process involving a large multi-disciplinary team spanning hospital and primary care (including the child’s general practitioner, for example). Although the leading role in diagnosis and the selection of therapeutic procedures falls on the oncologist, children and their parents meet frequently with a large multi-disciplinary team in different settings and for different reasons. Specialist nurses are integrated members of the multi-disciplinary team who are trained in child-centred communication and family support and yet their perspectives are largely absent from literature.

The conduct of research and recruitment of children with cancer at the terminal stage is acknowledged as being ethically and technically highly challenging. Nonetheless, the few young people who were consulted had as their goal a willingness to help other children by participating in research. The ethical challenges preventing inclusion of children and young people in research draw attention to the child as an inchoate participant in a social reality, rather than as an isolated and vulnerably entity, in which both protection of vulnerability and contribution to the good of children generally and society specifically ought be considered. Although technically challenging and varying in quality all of the studies were considered of reasonable quality to include in the review. All the studies, except three, were cross-sectional in design; and none of them reported sample size for quantitative studies or data saturation for qualitative studies thereby indicating the need for better designed and reported studies in the future.

Finally, key aspects of decision-making remain unexplored, such as information processing, voluntary nature (or not) of autonomous action and decisions made, and the ethical nature of medical and clinical work. More empirical work is therefore needed to find out how particular values, either professional or personal, manifest themselves in clinical practice. To research the relationship between individual and organizational ethical values (such as religious affiliation) would also help to explore the nature of decision-making in greater depth. Here again, interpersonal, comparative empirical research could reveal much about the similarities and differences between organizations and individual practice, and how these values interact in positive and negative ways on decision-making in this context.

## Conclusion

The findings of this review provide valuable, critical and new theoretical insights into how the decision-making processes are understood and constructed by the main actors when the decisions are of great consequence to the child and their family at the moment when treatment is no longer curative. It also provides impetus for more effective implementation of the ethics of professionalism in daily clinical practice thereby reinforcing the practice of good medicine. Ethical guidelines and regulations attempt to bring doctors together by articulating shared values. But, while important, ethics training must be supported by other institutional/organizational measures to assist doctors to achieve and maintain good professional standards. Findings will hopefully stimulate further normative and descriptive lines of research in this complex, under-researched, field through a wider cultural lens that includes children’s perspectives. The wider view will characterize and understand more fully the dynamics of the decision-making process in this specific end-of life context.

## Competing interests

The authors have declared that no competing interests exist.

## Authors’ contributions

EV and JN were responsible for review questions and design. EV conducted the electronic and hand search of the literature. EV and MB conducted the quality appraisal. EV and JN undertook the analysis, developed the analytical model and the theoretical framework. EV, JN and MB made critical revisions to the paper. JN supervised the study. All authors read and approved the final paper.

## Authors’ information

Dr Edith Valdez is a medical researcher. Her current areas of interest include medical ethics, research ethics, and clinical epidemiology.

Professor Jane Noyes is a health services researcher specializing in child health research, in the UK. She has a specific interest in systematic review methodology, and is Lead convenor of the Cochrane Qualitative Research Methods Group and Co-Chair of the Cochrane Methods Board Executive.

Dr Miguel Bedolla is a clinical ethics consultant and a teacher and researcher in the foundations of medical ethics.

## Pre-publication history

The pre-publication history for this paper can be accessed here:

http://www.biomedcentral.com/1471-2431/14/124/prepub

## Supplementary Material

Additional file 1 Quality assessment of included studies. Click here for file
