# Supplementary material for: When to stop? Decision-making when children’s cancer treatment is no longer curative: a mixed-method systematic review
Source: BMC Pediatr. 2014 May 13;14:124. doi: 10.1186/1471-2431-14-124 (PMC4028290; doi:10.1186/1471-2431-14-124)
Supplement: Additional file 1 — Quality assessment of included studies. [file 1471-2431-14-124-S1.docx]

| **Quality assessment of included qualitative studies** | | | | | | | |
| --- | --- | --- | --- | --- | --- | --- | --- |
| **Criteria** | **Study identification** | | | | | | |
|  | **33** | **34** | **35** | **36** | **37** | **38** | **39** |
| Was there a clear statement of the aims of the research? | 2 | 2 | 2 | 2 | 2 | 2 | 2 |
| Is a qualitative methodology appropriate? | 2 | 2 | 2 | 2 | 2 | 2 | 2 |
| Was the research design appropriate to address the aims of the research? | 2 | 2 | 2 | 1 | 1 | 2 | 1 |
| Was the recruitment strategy appropriate to the aims of the research? | 2 | 2 | 2 | 1 | 1 | 2 | 2 |
| Were the data collected in a way that addressed the research issue? | 1 | 1 | 1 | 1 | 1 | 1 | 1 |
| Has the relationship between researcher and participants been adequately considered? | 2 | 1 | 2 | 2 | 2 | 2 | 2 |
| Have ethical issues been taken into consideration? | 2 | 2 | 2 | 2 | 2 | 2 | 2 |
| Was the data analysis sufficiently rigorous? | 1 | 1 | 1 | 1 | 1 | 1 | 1 |
| Is there a clear statement of findings? | 2 | 1 | 2 | 1 | 2 | 2 | 2 |
| How valuable is the research? | 1 | 1 | 1 | 1 | 1 | 2 | 1 |
| **Total score/possible maximum score** | **17/20** | **15/20** | **17/20** | **14/20** | **15/20** | **18/20** | **16/20** |
| 2 = Yes; 1 = Partial; 0 = No | | | | | | | |

| **Quality assessment of included quantitative and mix-method studies (*continued*)** | | | | | | | | | | | |
| --- | --- | --- | --- | --- | --- | --- | --- | --- | --- | --- | --- |
| **Criteria** | **Study identification** | | | | | | | | | | |
|  | **22** | **23** | **24** | **25** | **26** | **27** | **28** | **29** | **30** | **31** | **32** |
| Was there a clear statement of the aims of the research? | 2 | 2 | 2 | 2 | 1 | 2 | 2 | 2 | 2 | 1 | 2 |
| Is the methodology appropriate? | 2 | 2 | 2 | 2 | 1 | 2 | 2 | 2 | 2 | 1 | 2 |
| Was the research design appropriate to address the aims of the research? | 2 | 2 | 2 | 1 | 1 | 1 | 2 | 1 | 2 | 1 | 2 |
| Was the recruitment strategy appropriate to the aims of the research? | 2 | 1 | 1 | 2 | 1 | 1 | 1 | 1 | 1 | 1 | 1 |
| Were the data collected in a way that addressed the research issue? | 1 | 1 | 1 | 1 | 1 | 1 | 1 | 1 | 1 | 1 | 1 |
| Has the relationship between researcher and participants been adequately considered? | 2 | 2 | 2 | 1 | 2 | 2 | 2 | 2 | 2 | 2 | 2 |
| Have ethical issues been taken into consideration? | 2 | 2 | 2 | 2 | 2 | 2 | 2 | 2 | 2 | 2 | 2 |
| Was the data analysis sufficiently rigorous? | 1 | 1 | 1 | 1 | 1 | 1 | 1 | 1 | 1 | 1 | 1 |
| Is there a clear statement of findings? | 2 | 2 | 2 | 2 | 2 | 1 | 2 | 2 | 1 | 2 | 2 |
| How valuable is the research? | 1 | 1 | 1 | 1 | 1 | 1 | 1 | 1 | 1 | 1 | 1 |
| **Total score/possible maximum score** | **17/20** | **16/20** | **16/20** | **15/20** | **13/20** | **14/20** | **16/20** | **15/20** | **15/20** | **13/20** | **16/20** |
| 2 = Yes; 1 = Partial; 0 = No | | | | | | | | | | | |
